# Regulation of Safracin Biosynthesis and Transport in *Pseudomonas poae* PMA22

**DOI:** 10.3390/md22090418

**Published:** 2024-09-13

**Authors:** J. Gerardo Hernández Delgado, Miguel G. Acedos, Fernando de la Calle, Pilar Rodríguez, José Luis García, Beatriz Galán

**Affiliations:** 1 Department of Biothecnology, Centro de Investigaciones Biológicas Margarita Salas, Agencia Estatal Consejo Superior de Investigaciones Científicas (CSIC), 28040 Madrid, Spain; gerardohdez88@gmail.com (J.G.H.D.); miguel.garciaacedos@ciemat.es (M.G.A.); jlgarcia@cib.csic.es (J.L.G.); 2 Research and Development Department, PharmaMar S.A., 28770 Madrid, Spain; fdelacalle@pharmamar.com (F.d.l.C.); prodriguez@pharmamar.com (P.R.)

**Keywords:** nonribosomal peptides, safracin, biosynthetic cluster genes (BGCs), transcriptional regulation, transporter genes

## Abstract

*Pseudomonas poae* PMA22 produces safracins, a family of compounds with potent broad-spectrum anti-bacterial and anti-tumor activities. The safracins’ biosynthetic gene cluster (BGC sac) consists of 11 ORFs organized in two divergent operons (*sacABCDEFGHK* and *sacIJ*) that are controlled by *P_a_* and *P_i_* promoters. Contiguous to the BGC sac, we have located a gene that encodes a putative global regulator of the LysR family annotated as MexT that was originally described as a transcriptional activator of the MexEF-OprN multidrug efflux pump in *Pseudomonas*. Through both in vitro and in vivo experiments, we have demonstrated the involvement of the dual regulatory system MexT-MexS on the BGC sac expression acting as an activator and a repressor, respectively. The MexEF-OprN transport system of PMA22, also controlled by MexT, was shown to play a fundamental role in the metabolism of safracin. The overexpression of *mexEF-oprN* in PMA22 resulted in fourfold higher production levels of safracin. These results illustrate how a pleiotropic regulatory system can be critical to optimizing the production of tailored secondary metabolites, not only through direct interaction with the BGC promoters, but also by controlling their transport.

## 1. Introduction

Nonribosomal peptides (NRPs) belong to a class of microbial secondary metabolites that have been a prolific source of bioactive compounds with a wide spectrum of medicinal applications, including antibiotics, immunosuppressants, and antineoplastics. This structurally diverse group is assembled from amino acid building blocks by a common thiotemplate-directed multistep reaction mechanism catalyzed by large modular enzymes termed nonribosomal peptide synthetases (NRPSs) [1,2]. Each module comprises domains for the incorporation of a single building block, typically involving an adenylation (A) domain for substrate recognition and activation, a thiolation (T) domain for covalent substrate tethering, and a condensation (C) domain for peptide bond formation. The building blocks of NRPs are not limited to the twenty proteinogenic amino acids. Unlike ribosomes, NRPSs can accept numerous possible monomers in their assembly line (>520 monomers, including rare nonproteinogenic amino acids), which makes them highly diverse. Additional product diversifications arise during the chain assembly, chain termination, and post-assembly line tailoring reactions [3].

Many specialized metabolites perform their ecological roles extracellularly and therefore require transport across cellular membranes. Transporter genes often colocalize in BGCs and have been shown to be compound-specific and necessary for export of the product in many cases [4,5,6]. The transport genes found in BGCs are used not only for the export of the final product or its intermediates from the cell, but also for the transport of intermediates between cell compartments [7,8,9]. Previous studies have shown that 56% of the bacterial BGCs in MIBiG 2.0 database of biosynthetic gene clusters contained at least one Pfam transporter domain [10]. Although some of the domains are specific for exporters, some of them can also be involved in the uptake of molecules like, for instance, precursor metabolites. However, a significant proportion of BGCs contain no transporter at all. A potential biological reason could be that the required transporter(s) may be encoded elsewhere in the genome.

**Figure 1:**
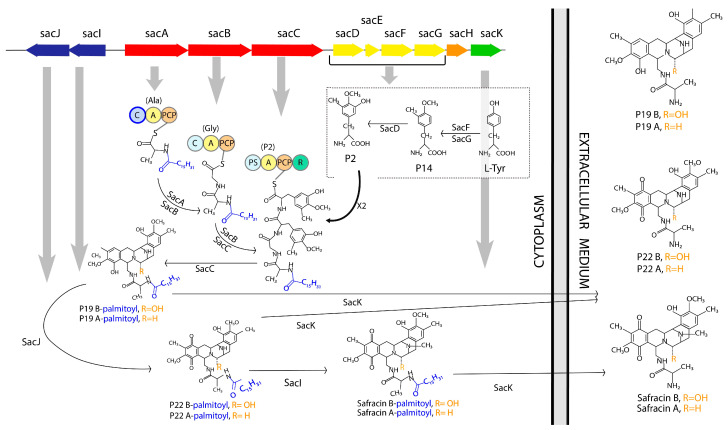
Proposed pathway for the biosynthesis of safracin. The *sacABCDEFGHK* and *sacIJ* operons; the modular organization of the domains of the SacA, SacB, and SacC NRPSs; and the main reactions of the biosynthetic pathway of safracin are illustrated. The indicated domains are: C, condensation domain; A, adenylation domain; PCP, peptidyl-carrier protein domain; R, reductase domain; and PS, condensation domain with the function of iteratively incorporating the P2 intermediate through the Pictet Spengler reaction. On each domain A, the amino acid that they specifically recognize and activate is specified in parentheses. The C-domain bordered by a blue line is proposed as an incorporator of palmitoyl, which is also indicated in blue on molecules along the biosynthetic pathway scheme. The final molecules identified in the extracellular medium represent the analogs Safracin B and A, whose difference is defined in the position R indicated in orange. Thick grey arrows relate each gene to the corresponding enzyme reaction.

*Pseudomonas poae* PAM22, formerly *Pseudomonas fluorescens* A2-2, was isolated from a soil sample collected in Tagawagun (Fukuoka, Japan) [11,12,13,14]. This bacterium has the ability to produce safracins, a family of compounds with potent broad-spectrum anti-bacterial and anti-tumor activities. Currently, the safracin B obtained by fermentation is modified to cyanosafracin B, which is used for the chemical synthesis of ET-743, an anti-tumor agent derived from the marine tunicate *Ecteinascidia turbinata*, which is active against various solid tumor cell lines [14]. The similarity observed between the structures of safracin and saframycin, an antibiotic produced by *Myxococcus xanthus* [15] and synthesized by a non-ribosomal peptide synthetase (NRPS), led to the identification of the safracin biosynthetic gene cluster (BGC sac) in *P. poae* PAM22 [14]. The BCG sac consists of 10 ORFs organized into two divergent operons (*sacABCDEFGH* and *sacIJ*), which cover 17.5 kb and are flanked by ORFs encoding putative transposase fragments (Figure 1). Through comparative analyses, it was suggested that the *sacABCDEFGH* operon is responsible for the safracin skeleton, whereas the *sacIJ* operon is responsible for the final tailoring of safracins. A recent study confirmed the participation of a cryptic palmitoyl fatty acyl chain in the biosynthesis of safracin, forming a prodrug that is preferred by the tailoring enzymes SacI and SacJ [16]. Post-NRPS modification and maturation steps consist of the A-ring oxidation of compound P19 by the FAD-dependent monooxygenase SacJ, generating the analogs P22B and P22A (Figure 1), which are subsequently methylated by SacI. After the A-ring oxidation and subsequent N-methylation, the fatty acyl chain is removed by the membrane-bound protein SacK [16].

This finding established a new boundary in the BGC sac 3′ end at *sacK* gene. On the other hand, the limit of the BGC sac at the 5′ end has not been determined to date, and the presence and role of putative regulatory proteins of the cluster have not been investigated. In this sense, we observed that, contiguous to the 5′ end of BGC sac, there is a regulatory gene, *IMF22_10365*, encoding a protein homologous to the transcriptional regulator MexT from *Pseudomonas aeruginosa* (Figure 1). MexT is a global LysR transcriptional regulator (LTTR) known to modulate antibiotic resistance and virulence in *P. aeruginosa*. It was originally described as a transcriptional activator of the MexEF-OprN multidrug efflux pump, which confers resistance to chloramphenicol, trimethoprim, and fluoroquinolones and negatively affects the expression of the coding genes of the efflux pumps MexAB-OprM and MexXY/OprM, making the bacterium sensitive to certain antibiotics such as β-lactams and aminoglycosides [17,18]. In the *P. aeruginosa* genome, *mexT* is located contiguous to *mexEF-oprN*, but in most species of *Pseudomonas*, this gene is found in different loci and distant from this transport system, which is considered an indication of the pleiotropic role of this regulator [19,20]. The MexT regulator consists of 305 aa, with an N-terminal DNA-binding domain and a C-terminal regulatory domain (RD), and interacts with the target promoter regions by joining two nod-box operator boxes with the consensus sequences ATYA(N7)YGAT and ATYA(N7)YGAT(N4) [21]. The adjacent gene, named *mexS*, encodes a putative oxidoreductase, and its activity causes a repressive function of the MexT regulator. Despite the many studies carried out on MexT, the molecular bases and the nature of the co-inductor ligand that allow its activation are still unknown. On the other hand, the vast majority of studies have focused on *P. aeruginosa*, relating MexT function with oxidative stress response processes and activation of virulent phenotypes due to the pathogenic nature of this species. However, to the best of our knowledge, the role of MexT in the biosynthesis of natural products in environmental bacteria has not been reported.

In this work, we analyze the roles of the *IMF22_10365* and *IFM22_10360* genes coding the putative MexT-MexS regulatory system of *P. poae* PMA22 in safracin biosynthesis. We show how safracin biosynthesis and its transport to the extracellular medium is nicely coordinated by this dual regulatory system through the transcriptional activation of the BGC sac tailoring genes (*sacI* and *sacJ*) and the MexEF-OpnR multidrug efflux pump.

## 2. Results

### 2.1. Co-Localization of mexT and BGC Sac in P. poae PMA22 Genome

**Figure 2:**
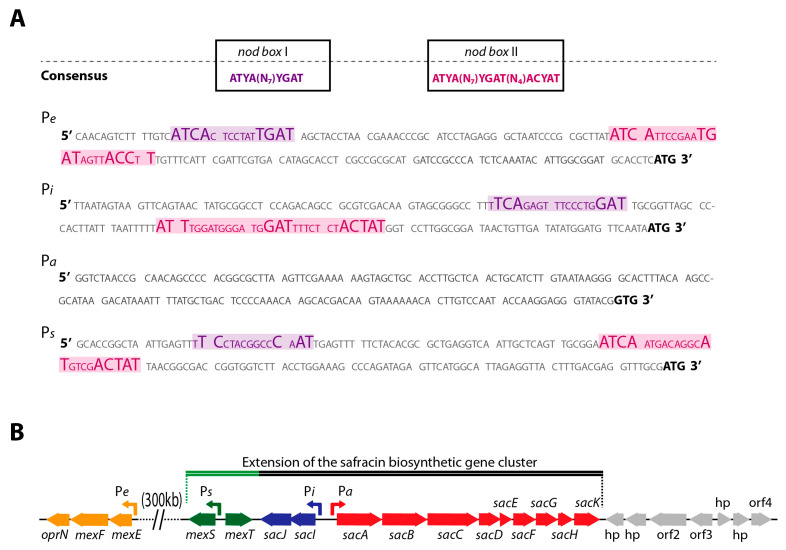
Putative promoters involved in the biosynthesis of safracin. (**A**) The sequences of the BGC sac promoters (*P_a_* and *P_i_*), the *mexEF-oprN* transport system promoter (*P_e_*), and the *mexS* promoter (*P_s_*) are detailed. The two nod box consensus sequences described in the literature are indicated. For each promoter, the sequences identified as nod box I and nod box II are highlighted in color and the nucleotides coinciding with the consensus nod-box sequences are enlarged. (**B**) Diagram of the genetic organization of the BGC sac and its surrounding environment. The diagram includes the close regulatory system *mexT* and *mexS* and the *mexEF-oprN* transport system operon, located at 300 kb.

To localize regulatory genes for the BGC sac, its genome environment was examined in silico. Contiguous to the BGC sac, there is the *IMF22_10365* gene encoding a possible regulator of the LysR family. This gene is annotated as *mexT* because the encoded protein shows 98.62% identity with MexT from *P. aeruginosa*. Contrary to what happens in *P. aeruginosa*, *mexT* is located in the *P. poae* PMA22 genome 300 kb away from the *mexEF*-oprN genes encoded by *IMF22_09105*, *IMF22_09100*, and *IMF22_095* genes, respectively, in *P. poae* PMA22 (Figure 2A). Interestingly, the two possible MexT DNA binding sites in the *P_e_* promoter region of *mexEF-oprN* are also present in the corresponding region of *P. poae* PMA22 (Figure 2B).

The proximity of *mexT* to the BGC sac of *P. poae* PMA22 suggested a possible regulatory role on the biosynthetic pathway of safracin. The analysis of the BGC sac promoter regions (named *P_i_* and *P_a_*) showed two nod-boxes at *P_i_* promoter, but no nod-box was found within the *P_a_* promoter (Figure 2A). This suggests that MexT could play a regulatory role in safracin production by controlling the expression of the tailoring enzymes whose expression is driven by the *P_i_* promoter. The MexS protein encoded by *IMF22_10360* gene, located adjacent to the *mexT* gene, shares 59.05% sequence similarity with MexS from *P. aeruginosa*. The EMBL Pfam tool suggests that MexS may have alcohol dehydrogenase (ADH) activity and predicts a catalytic zinc-binding domain between residues 28 and 90, as well as a cofactor-binding domain between residues 162 and 272. Although the interaction of MexT on the *mexS* promoter region (P_s_) has been demonstrated in *P. aeruginosa*, the sequences of the nod-box have not been identified [22], and it was assumed that MexT from *P. aeruginosa* could bind DNA sequences lacking a nod-box [17,19]. However, we found a nod-box sequence at *P_s_* promoter of *mexS* from *P. poae* PMA22 (Figure 2B), strongly suggesting the involvement of the dual regulatory system MexT-MexS on the BGC sac expression. The confirmation of this hypothesis will expand the BGC sac cluster boundaries at the 5′ end (Figure 2A).

### 2.2. MexT and MexS Are Functional in P. poae PMA22

The *mexT* and *mexS* genes are considered mutational “hot spots” in *P. aeruginosa*, and their sequence diversity is used as a predictor of PAO1 lineage integrity in laboratory strains [22,23]. For this reason, the utilization of strains carrying mutations in the *mexT* gene could negatively affect the reproducibility of the results and complicate their interpretation. In many cases, the mutations which were found resulted in a total loss of MexT and MexS function [22,23,24,25]. It is common to isolate *P. aeruginosa* strains that have a non-functional MexT regulator because the mutants have the advantage of accelerated growth during the early exponential phase, and they outgrow wild-type cells in a mixed population [25]. On the other hand, it has been described that a non-functional MexS mutant is sufficient to cause the phenotype called *nfxC* in *P. aeruginosa*. The *nfxC* mutants have the MexEF-OprN multidrug efflux pump strongly induced and acquire resistance to chloramphenicol, trimethoprim, and fluoroquinolones. The underlying mechanisms that give rise to nfxC mutants are not fully known, but appear to be multifactorial [22,25,26].

**Figure 3:**
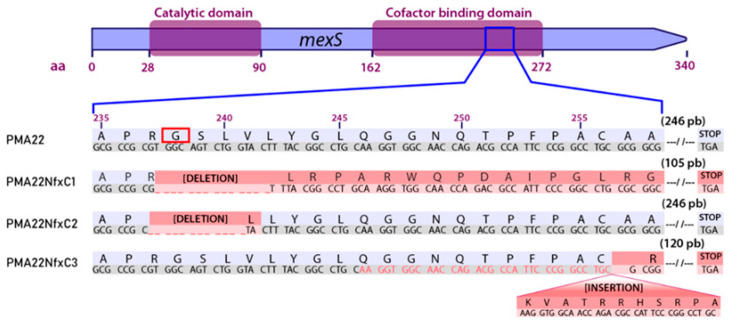
MexS sequences of *P. poae* PMA22 with *nfxC* phenotype. A) The functional domains of MexS are indicated according to the predictions of the Pfam algorithm. The nucleotide and amino acid sequences of MexS from wild-type and mutant strains are shown. The red box on the sequence of MexS indicates the highly conserved Gly residue in alcohol dehydrogenase-type enzymes. The shaded regions indicate the sequences affected by phase shifts in the codon readout, caused by deletions or insertions. In the sequences corresponding to the PMA22NfxC3 mutants, the nucleotides written in red are those that are duplicated in the insertion sequence that follows them.

Considering these facts, it is not possible to deduce only based on the MexT and MexS sequence whether these proteins are functional in *P. poae* PMA22, and therefore, we performed a previous validation of the functionality of both proteins. To determine the role of the MexT-MexS system in *P. poae* PMA22, we have isolated and characterized three spontaneous mutants with the *nfxC* phenotype, called PMA22NfxC1, PMA22NfxC2, and PMA22NfxC3 (see Section 3). The sequencing of the *mexT* and *mexS* genes in these strains revealed that the *nfxC* phenotype occurs due to mutations in the *mexS* gene, specifically within the binding domain of the MexS cofactor, i.e., between aa 236 and 257 (Figure 3). The *nfxC* phenotype on PMA22NfxC1 and PMA22NfxC2 strains is caused by a deletion of 14 bp and 12 bp, respectively, at residue 237 (Figure 3). The PMA22NfxC1 strain contains a 14 bp deletion that changes the reading frame, causing a premature stop codon. The 12 bp deletion found in the PMA22NfxC2 strain rendered a 3 aa deletion and a 1 aa substitution (R23xL) (Figure 3). The Gly-deleted residue in the protein is highly conserved, along with the position of other seven Gly residues, in ADHs of organisms belonging to different kingdoms (mammals, plants, and bacteria) [27,28], suggesting that this deletion may be the major cause of MexS dysfunction. On the other hand, the PMA22NfxC3 strain has a duplication of 32 bp after the encoded residue 256, which leads to the appearance of a premature stop codon (Figure 3). Since the PMA22NfxC1 strain has a premature stop codon in the *mexS* sequence and, consequently, a clear nonfunctional MexS protein, we decided to use this strain as a control reference in subsequent experiments. 

To demonstrate MexS functionality in the wild-type PAM22 strain and validate the *nfxC* phenotype in PMA22NfxC1, they were transformed with the pmexS plasmid, generating PAM22 (pmexS) and PMA22NfxC1 (pmexS) strains. They were cultivated in LB plates containing Cm (600 µg/mL) to compare the frequency of the generation of mutants between them. When using the PMA22NfxC1 (pmexS) strain, the mutation frequency was 5.84 × 10^−9^, which was only an order of magnitude lower than the wild-type strain PMA22, which was 1.09 × 10^−8^, suggesting that the complemented strain PMA22NfxC1 (pmexS) recovered the wild-type phenotype after supplementation, and, more importantly, that MexS is functional in the wild-type strain. This result shows that the selection pressure to generate *nfxC* mutants mainly compromises the *mexS* gene.

It has been described that the maintenance of MexT functionality is essential for the generation of *nfxC* mutants in *P. aeruginosa* [21]. Here, we obtained similar results with *P. poae* PMA22 strain since no mutations in the *mexT* gene sequence were detected in any of the isolated nfxC mutants. This result might confirm the functionality of MexT in the *P. poae* PMA22 strain.

### 2.3. In Vitro Interaction of MexT with P_a_ and P_i_ Promoters

**Figure 4:**
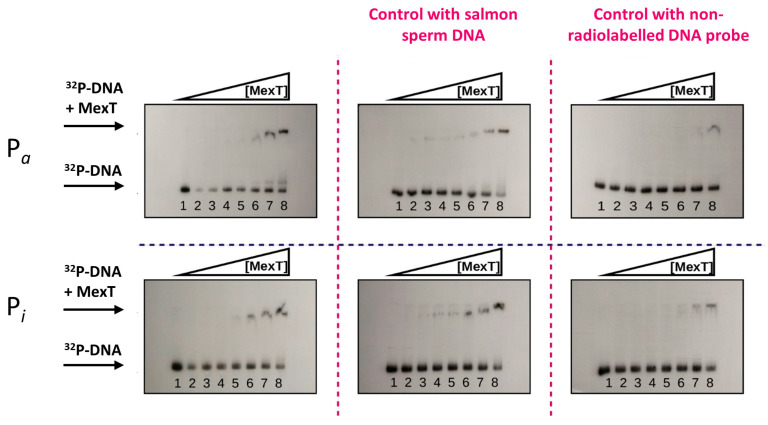
EMSA assays of MexT and *P_i_* and *P_a_* promoters. EMSA assays were performed using increasing concentrations of MexT protein. Lane 1 to 8: 0, 5.4, 6.7, 9.0, 13.4, 26.9, 53.8, and 134.5 ng of MexT. EMSA control assays were performed using 50 µg/mL of salmon sperm DNA (second column) or 50 µg/mL of the corresponding non-radiolabeled probe (third column).

To study the physical interaction of MexT with the P_i_ and P_a_ promoters, the MexT protein was overproduced by cloning the *mexT* gene in the pET29 expression vector, generating the pET29mexT plasmid that allows its production in the *Escherichia coli* BL21 (pET29mexT) strain as a fusion protein with a 6His-tag (MexT-(His)_6_) (Figure S2). When the interaction of purified MexT with *P_i_* and *P_a_* promoters was studied by gel retardation assays (EMSAs), we observed that MexT-(His)_6_ interacted with both promoters, although the affinity of MexT-(His)_6_ for the Pi probe was approximately threefold higher than for the Pa probe (Figure 4). To demonstrate the specificity of MexT-(His)_6_ binding to Pa and Pi probes, EMSAs were performed in the presence of salmon sperm DNA and increased concentrations of unlabeled Pa and Pi probes as controls. While salmon sperm DNA did not affect the binding of MexT-(His)_6_ to Pi and Pa probes, the addition of unlabeled DNA Pi and Pa fragments abolished the interaction of MexT, suggesting that the binding of MexT-(His)_6_ to Pa and Pi probes is specific (Figure 4). These results suggest that the MexT/MexS regulatory system could play a role in the transcription of the genes driven by the *P_i_* and *P_a_* promoters and, therefore, in the biosynthesis of safracin.

### 2.4. Role of MexT in the Expression of BGC Sac

**Figure 5:**
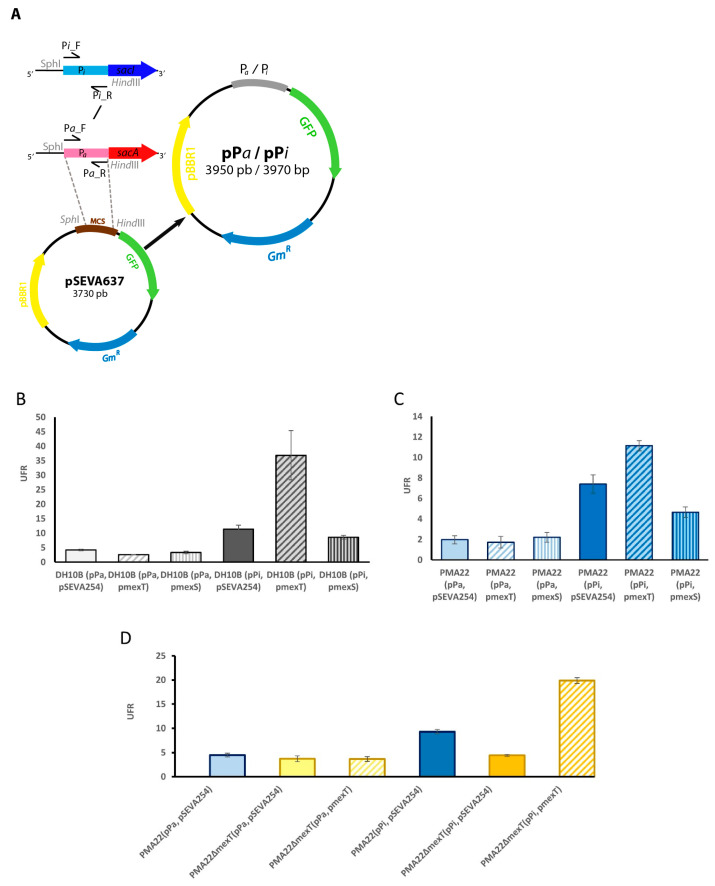
Analysis of *P_i_* and *P_a_* promoters in vivo in the presence of MexT or MexS regulators. (**A**) Construction of pPa and pPi plasmids in which *P_a_* and *P_i_* promoters are transcriptionally fused to the *gfp* reporter gene in plasmid pSEVA637. (**B**) Expression of *P_i_* and *P_a_* promoters in *E. coli* DH10B. (**C**) Expression of *P_i_* and *P_a_* promoters in *P. poae* PMA22. (**D**) Expression of *P_i_* and *P_a_* promoters in *P. poae* PMA22ΔmexT. Experiments were performed in MS medium supplemented with 4% mannitol. The promoter activities were determined after 24 h of culture and are represented in relative fluorescence units (RFU).

To investigate the role of MexT in vivo, we constructed pPa and pPi plasmids carrying a transcriptional fusion of *P_a_* and *P_i_* with the *gfp* reporter gene, respectively (Figure 5A). These plasmids allowed us to measure the expression of BGC sac genes in different mutant strains and to select the best expression conditions by measuring the fluorescence of the GFP protein. To study the role of MexT and MexS regulators, pPa y pPi plasmids were transformed in *E. coli* DH10B, yielding *E. coli* DH10B (pPa) and *E. coli* DH10B (pPi) strains, respectively. Next, these strains were co-transformed with pmexT, pmexS, and pSEVA254 plasmids, and the resulting strains (i.e., DH10B (pPa, pmexT), DH10B (pPa, pmexS), DH10B (pPa, pSEVA254), DH10B (pPi, pmexT), DH10B (pPi, pmexS), and DH10B (pPi, pSEVA254), were cultured in the safracin production MS medium with 4% mannitol. *P_i_* promoter rendered higher expression levels of *gfp* than the *P_a_* promoter in *E. coli* (Figure 5B). Moreover, the overexpression of MexT and/or MexS did not have any effect on the gfp expression driven by *P_a_*, which was expressed at low levels. Nevertheless, we observed significant changes in the *gfp* expression driven by P_i_. The *E. coli* DH10B (pPi, pmexT) strain showed higher GFP expression levels, while the DH10B (pPi, pmexS) strain showed lower expression levels of *gfp* when compared to those of the DH10B (pPi, pSEVA254) control strain (Figure 5B).

These plasmids were transformed in the native *P. poae* PAM22 strain as well, yielding PMA22 (pPa, pmexT), PMA22 (pPa, pmexS), PMA22 (pPa, pSEVA254), PMA22 (pPi, pmexT), PMA22 (pPi, pmexS), and PMA22 (pPi, pSEVA254) strains, which were cultured in MS medium in the presence of 4% mannitol. The studies performed using these strains showed that the overexpression of *mexT* and *mexS* genes had a significant effect on the GFP expression driven by the *P_i_* promoter, increasing its expression in the presence of MexT and diminishing in the presence of MexS (Figure 5C). However, they did not affect the GFP expression driven by *P_a_*. These findings agree with the results observed in *E. coli*.

To further characterize the function of MexT, we deleted the *mexT* gene in PAM22. The resulting strain, named PMA22ΔmexT, was transformed with pPa, pPi, pSEVA254, and pmexT plasmids, yielding the PMA22ΔmexT (pPa, pSEVA54), PMA22ΔmexT (pPa, pmexT), PMA22ΔmexT (pPi, pSEVA254), and PMA22ΔmexT (pPi, pmexT) strains, which were used to monitor the GFP expression driven by *P_a_* and *P_i_* promoters in the safracin-producing medium. The fluorescence intensity observed in the strains carrying the *P_a_* promoter confirmed that MexT did not have a role in the GFP expression driven by this promoter under the tested conditions (Figure 5D). However, the deletion of *mexT* affected the GFP expression driven by *P_i_* promoter, since the PMA22ΔmexT (pPi, pSEVA254) strain showed two-fold lower fluorescence intensity than the PMA22 (pPi, pSEVA254) strain. After complementation with the plasmid pmexT, the fluorescence intensity of PMA22ΔmexT (pPi, pmexT) increased dramatically, confirming the MexT activator’s role (Figure 5D).

Taken together, all these data support our hypothesis that MexT and MexS act as an activator and a repressor, respectively, on the expression of BGC sac-tailoring enzymes.

### 2.5. Effect of MexT and MexS on Safracin Production

**Figure 6:**
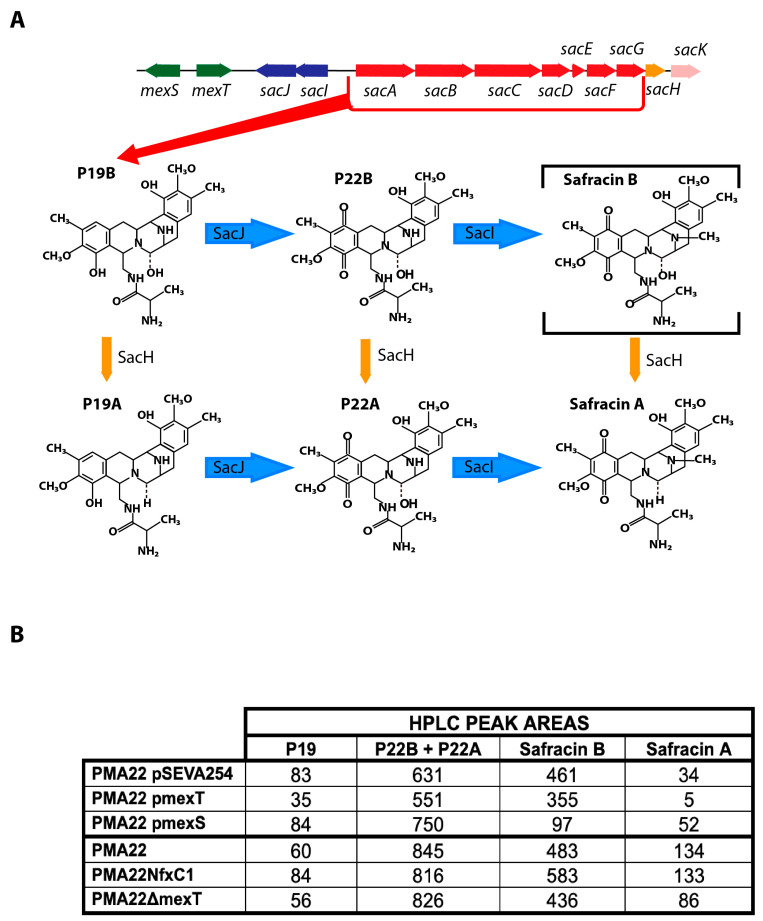
Effects of MexT and MexS on safracin and intermediate metabolite production. (**A**) Scheme of the chemical reactions performed by SacJ, SacI, and SacH tailoring enzymes. (**B**) HPLC peak areas of intermediates P19A, P19B, P22A, and P22B and final products safracin A and safracin B delivered by the different tested strains.

The *P_i_* promoter drives the expression of the tailoring genes *sacI* and *sacJ*, encoding a methyltransferase and a monooxygenase, respectively. SacJ oxidizes the analog P19B, generating the compound P22B, which is methylated by SacI, delivering safracin B. In addition, P19B, P22B, and safracin B can be hydroxylated and transformed in P19A, P22A, and safracin A, respectively, by SacH (Figure 1 and Figure 6A). Therefore, to study the effects of MexT and MexS on safracin production through the control of the *P_i_* promoter, it was necessary to identify the aforementioned analogues of the biosynthetic pathway. For this purpose, we used a bioreactor culture supernatant highly enriched in safracin and safracin analogues (kindly provided by Pharmamar) as a reference, which was analyzed by HPLC-MS (Figure S2A). The production of safracin analogues was analyzed in the PMA22, PMA22NfxC1, and PMA22ΔmexT strains, as well as in the recombinant PMA22 (pmexT), PMA22 (pmexS), and PMA22 (pSEVA254) strains. The overexpression of *mexS* in PMA22 (pmexS) led to a significant decrease in safracin production and an increase in the concentrations of P22A and P22B intermediates when compared to PMA22 (pSEVA254), i.e., the wild-type strain carrying the empty plasmid (Figure 6B and Figure S2B). This result further supports the role of MexS as a repressor. However, we did not observe an effect on safracin production when mexT was overexpressed in PMA22 (pmexT). Nevertheless, the PMA22ΔmexT strain showed slightly lower production of safracin and higher accumulation of P19, P22A, and P22B analogs when compared to the wild-type strain PMA22 (Figure 6B and Figure S2B). This result supports the hypothesis that the lower production of the tailoring enzymes SacJ and SacI, most likely because the *P_i_* promoter is not fully activated under these conditions, promotes the accumulation of P19 and P22 analogs. The analysis of the safracin production of PMA22NfxC1, harboring an inactivated MexS protein, showed that metabolic flow was channeled more efficiently to the production of safracin, resulting in a higher safracin production, but similar amounts of P19 and P22 when compared to PAM22 (Figure 6 and Figure S2B). These results further reinforce the activating and repressing roles proposed for MexT and MexS, respectively, in the production of safracin.

### 2.6. Implication of MexEF OprN Efflux Pump in the Transport of Safracin

**Figure 7:**
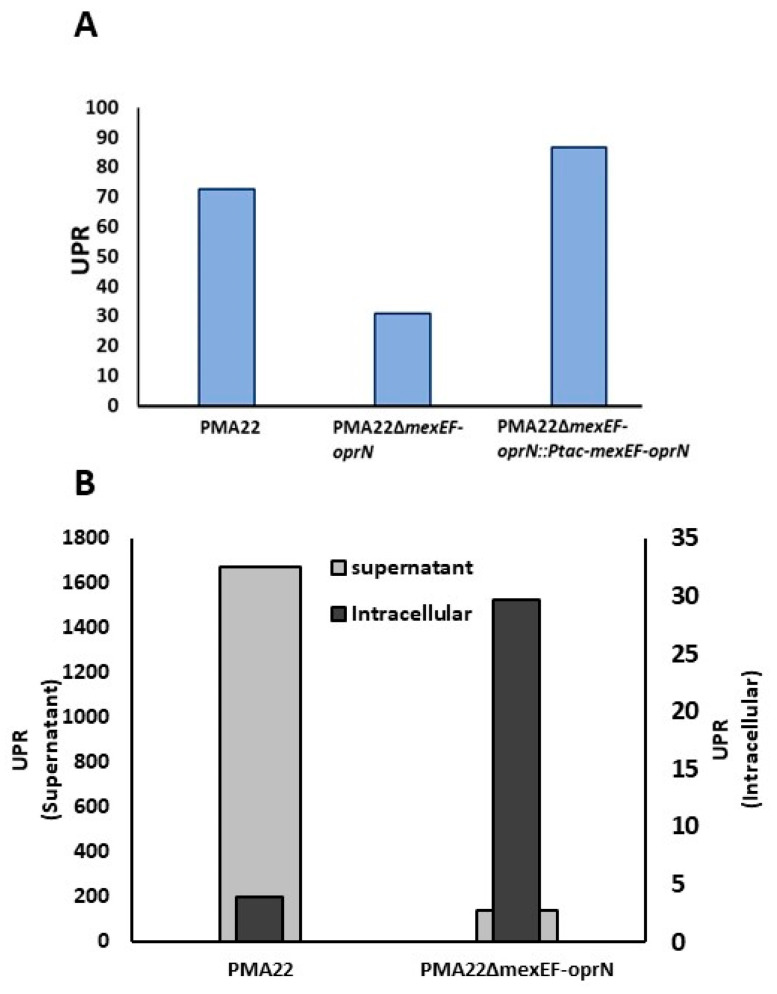
Effect of MexEF-OprN transport system on safracin production. (**A**) Safracin production expressed in relative production units (RPU) determined in M63P medium with 8% mannitol after 72 h of culture. (**B**) Safracin content in RPUs in the culture medium (left axis) and the cytoplasm (right axis) of PMA22 and PMA22ΔmexEF-oprN strains determined in M63P medium with 4% mannitol after 72 h of culture.

MexT was described as an activator of the MexEF-OprN transport system in *P. aeruginosa* PAO1, but, in addition, our data suggest that MexT is involved in the transcriptional activation of the *sacIJ* operon responsible for safracin tailoring. Therefore, MexT could coordinate the production of safracin and its transport to the extracellular medium through the MexEF-oprN efflux pump. To demonstrate the participation of MexEF-OprN in the transport of safracin, we constructed the strain PMA22ΔmexEF-oprN, in which this transport system was deleted (see materials a methods). PMA22 and PMA22ΔmexEF-oprN strains were cultivated in safracin-producing medium, and the safracin production was quantified by HPLC-DAD. We observed that, in the PMA22ΔmexEF-oprN strain, the amount of safracin detected in the supernatant was 11.5 times lower when compared to PMA22 than in the wild strain PMA22 cultivated under the same conditions (Figure 7A). When intracellular safracin production was measured in the same cultures, the inverse scenario was observed: the PMA22ΔmexEF-oprN strain contained 7.7 times more safracin than the wild PMA22 strain (Figure 7B), suggesting that MexEF-OprN efflux pump is involved in the metabolism of safracin. On the other hand, when the strains were cultivated in M63P medium with 4% mannitol, a similar result was obtained, although the production of the PMA22ΔmexEF-oprN strain was only 2.4 times lower than that obtained with the PMA22 strain (Figure 7B). In addition, to complement the PMA22ΔmexEF-oprN mutant, the *mexE*, *mexF*, and *oprN* genes were cloned in plasmid pGB (Table S2), generating the pBG_Ptac_mexEF_oprN plasmid, in which the expression of the MexEF-OprN transport system is driven by the *P_tac_* promoter and flanked by the sequences Tn7L and Tn7R from Tn7 transposon. This suicide plasmid was used to integrate Ptac-mexEF-oprN into the PMA22ΔmexEF-oprN genome, yielding the PMA22ΔmexEF-oprN::Ptac-mexEF-oprN strain.

As expected, the safracin production obtained with the complemented mutant strain PMA22ΔmexEF-oprN::Ptac-mexEF-oprN was similar to that obtained with PMA22 (Figure 7A). These results support the hypothesis that the MexEF-OprN efflux pump is most likely involved in the transport of safracin to the extracellular medium, and therefore, it is an essential element in the biosynthesis process.

**Figure 8:**
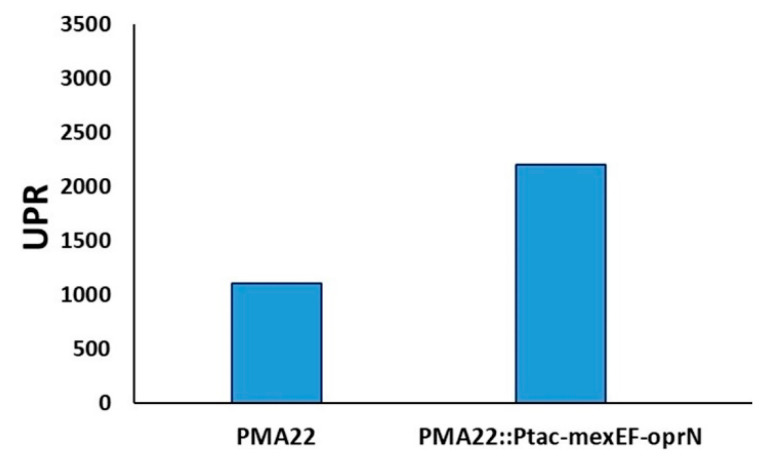
Production of safracin by over-expressing the MexEF-OprN transport system. Safracin production, expressed in relative production units (RPU), was determined in the culture medium of PMA22 and PMA22::Ptac-mexEF-oprN strains cultured in M63P medium with 4% mannitol and 0.02% casamino acids after 72 h of culture.

To reinforce the implication of the MexEF_OprN efflux pump in the metabolism of safracin, we hypothesized that its over-expression could increase safracin production. Therefore, we added an extra copy of the mexEF-oprN operon to the genome of PMA22, generating a new recombinant strain named PMA22::Ptac-mexEF-oprN. The over-expression of the MexEF-OprN transport system in the PMA22::Ptac-mexEF-oprN strain resulted in fourfold higher production of safracin compared to the PMA22 strain (Figure 8), confirming that the secretion of safracin plays a critical role in the biosynthesis process.

## 3. Materials and Methods

### 3.1. Bacterial Strains, Plasmids, Primers, Media, and Growth Conditions

The strains, plasmids, and primers used in this work are shown in Tables S1–S3. *Escherichia coli* strains were grown in LB medium at 37 °C with orbital shaking at 200 rpm, and in LB agar plates for solid media. LB agar plates were used to maintain *P. poae* PAM22 and the recombinant strains. Safracin production was achieved using rich and minimum media. Per liter, MS broth contains: 14 g (NH_4_)_2_SO_4_; 0.3 g K_2_HPO_4_; 0.1 g FeCl_3_.6H_2_O; 40 g mannitol; and yeast extract from 35 g whole yeast (Sensient). Yeast extract was prepared by mixing 7 g of yeast in 40 mL of distilled water in a 50 mL falcon tube. Then, the yeast cells were subjected to sonication (3 cycles of 5 min at maximum intensity in a Branson 150 sonicator) and centrifuged at 3800 rpm for 10 min at 4 °C using a Multispeed Centrifuge CL31R (Thermo Scientific) centrifuge. The solid fraction was discarded and the liquid fraction (extract) was kept at −20 °C until use. M63P medium is a modification of the M63 mineral medium and contains, per liter: 136 g KH_2_PO_4_; 20 g (NH_4_)_2_SO_4_; 0.25 g MgSO_4_; 0.005 g FeSO_4_·7H_2_O, 0.02% casamino acids; and trace elements whose composition per liter is: 2.78 mg FeSO_4_·7H_2_O; 1.98 mg MnCl_2_·4H_2_O; 2.81 mg CoSO_4_·7H_2_O; 1.47 mg CaCl_2_·2H_2_O; 0.17 mg CuCl_2_·2H_2_O; 0.29 mg ZnSO_4_·7H_2_O. As a carbon source, 4% (*w*/*v*) of mannitol was used. For some specific experiments, other carbon sources besides mannitol were used at a final concentration of 4% (*w*/*v*). When necessary, antibiotics were used at the following concentrations: kanamycin (Km) (50 μg/mL), gentamycin (Gm) (10 μg/mL), chloramphenicol (Cm) (12.5 μg/mL), and tetracycline (Tc) (7 μg/mL). When needed, 1 mM isopropyl-β-D-1-tiogalactopiranoside (IPTG) was added unless otherwise stated. *P. poae* strains were grown overnight in falcon tubes in the production medium at 30 °C with shaking at 200 rpm. The overnight culture was washed in 0.85% NaCl solution and diluted to an optical density (OD_600_) ≈ 0.12 in 20 mL of fresh medium. To determine the production of safracins, the strains were cultured for 72 h.

### 3.2. DNA Manipulation

DNA manipulation protocols were performed as described elsewhere [29]. Plasmid DNA was purified using a High Pure Plasmid Isolation Kit (Roche, Basel, Switzerland). DNA fragments were purified with the QIAquick PCR Purification Kit (Qiagen, Hilden, Germany) or QIAquick Gel Extraction Kit (Qiagen). *E. coli* cells were transformed using the RbCl method [29] or by electroporation using a Gene Pulser (Bio-Rad, Berkeley, CA, USA) [30]. DNA amplification was performed in a Mastercycler Gradient (Eppendorf, Hamburg, Germany) using the oligonucleotides listed in Table S3, which were purchased from Sigma-Aldrich (Merck KGaA, Darmstadt, Germany). Phusion High-Fidelity DNA Polymerase (New England Biolabs, Ipswich, MA, USA) was used for cloning amplifications and *Taq* DNA polymerase (Biotools, Madrid, Spain) for screening. PCR products were checked by agarose gel electrophoresis, and those aimed for cloning were confirmed by DNA sequencing using Secugen S.L. (Madrid, Spain). Digestion of DNA fragments was carried out using restriction enzymes (New England Biolabs), and ligation was performed with Instant Sticky-end Ligase Master Mix (New England Biolabs).

To facilitate the study of BGC sac expression under different culture conditions, we constructed a tool for the study of transcriptional expression based on the *gfp* reporter gene. For this purpose, the 220 pb DNA fragment upstream of the *sacA* gene, containing the putative *P_a_* promoter, as well as a 240 bp DNA fragment upstream *sacI* gene containing the putative *P_i_* promoter, were PCR-amplified with primers Pa_F/Pa_R and Pi_F/Pi_R (Table S3), digested with *Sph*I-*Hin*dIII, and cloned into the pSEVA637 plasmid (Table S2). The resulting pPa and pPi plasmids carried a transcriptional fusion of the corresponding promoter with *gfp*. Both plasmids were used as tools to study the expression of the BGC sac indirectly through the measurement of fluorescence due to *gfp* expression in the *E. coli* and *P. poae* strains.

To construct the pmexT plasmid, the *mexT* gene was PCR-amplified using mexT_F and mexT_R primers (Table S3), digested with *Xba*I-*Hin*dIII, and cloned into pSEVA254 (Table S2). In the same way, the *mexS* gene was PCR-amplified with mexS_F and mexS_R primers (Table S3), digested with *Eco*RI and *Xba*I, and cloned into pSEVA254 to deliver the pmexS plasmid.

### 3.3. MexT Overproduction and Purification

The MexT protein was heterologously produced with an extension of 6 histidines at its C-terminal end to facilitate further purification [21]. For the overproduction of MexT-(His)_6_, the *mexT* gene of *P. poae* PMA22 was PCR-amplified with the oligonucleotides pETmexT_F and pETmex_R, yielding pET29mexT (Table S2, Figure S1), then digested with *Nde*I-*Hin*dIII and cloned in the expression vector pET29a(+). Overproduction was carried out with the *E. coli* strain BL21 (DE3) containing the pET29mexT plasmid. All cultures were carried out in LB medium with Km incubated in agitation at 37 °C. From a preinoculum cultivated for 16 h, 100 mL of medium was inoculated at an initial OD_600_ of 0.05 and incubated at 37 °C. Once the culture reached an OD_600_ of 0.5, 0.05 mM IPTG was added and the incubation temperature was reduced to 25 °C. The culture was incubated for 4 h, and subsequently, the cells were collected by centrifugation and preserved at −20 °C.

Cells were resuspended in 10 mL of phosphate buffer (50 mM NaH_2_PO_4_, 50 mM Na_2_HPO_4_, 300 mM NaCl, 20 mM imidazole, pH 7.8). The cells were lysed at 1000 psi using a French Pressure Cell Press (American Instruments Company, Sao Paulo, Brasil). The extract obtained was centrifuged in a Laborzentrifugen 1–15K (Sigma-Aldrich, Saint Louis, MO, USA) at 4 °C and 20,000× *g* for 30 min, and the supernatant (raw extract) was collected, discarding the non-soluble fraction. Purification of the MexT-(His)_6_ protein was performed using the Ni-NTA spin kit (Qiagen). The protein elution was performed using an imidazole concentration gradient starting at 50 mM and reaching 2 M. Protein concentration was estimated spectrophotometrically at 280 nm, considering the theoretical molar extinction coefficient of the protein (15,470 M^−1^ cm^−1^). Aliquots showing higher absorbance at 280 nm were analyzed using SDS-PAGE (Figure S1). Fractions containing His6-MexT were dialyzed at 4 °C in the analysis buffer (20 mM Tris-HCl, 300 mM NaCl, 5% glycerol, 2 mM EDTA, pH 8.5). Subsequently, the protein was distributed in aliquots and stored at −20 °C.

### 3.4. Electro-Mobility Shift Assays (EMSA)

Two 240 bp and 220 bp DNA probes containing *P_i_* and *P_a_* promoters were generated by releasing two fragments of the pPi and pPa plasmids (Table S2). Plasmids had previously been purified using the Qiagen Plasmid Midi kit and digested with *Eco*RI and *Hin*dIII. The probes released after digestion were purified using the QIAquick Gel Extraction Kit (Qiagen) and labeled [α-^32^P]dATP (6000 Ci/mmol; Perkin-Elmer Life Sciences, Shelton, CT, USA) using the “Klenow fragment” of *E. coli* DNA polymerase I (5 U/µL, Promega, Fitchburg, WI, USA). The labeled fragments were purified using the QIAquick PCR Purification Kit (Qiagen). For titration assays, increasing amounts of purified MexT were incubated with 1 nM of the labeled probe in binding buffer (20 mM Tris-HCl at pH 8, 150 mM KCl, 10 mM MgCl_2_, 10% glycerol, 2 mM ß-mercaptoethanol and 50 μg/mL BSA) in a final volume of 9 μL. For specific and non-specific competition reaction mixtures, 10-, 100-, or 1000-fold excesses of the unlabeled probe and 0.5 μg, 1 μg, and 2 μg of unspecific DNA (salmon sperm) were added, respectively. The EMSA reaction mixtures were incubated for 20 min at room temperature and fractionated by electrophoresis on 5% polyacrylamide gels buffered with 0.5× TBE (45 mM Tris-borate, 1 mM EDTA). The gels were dried onto Whatman 3MM paper and exposed to Hyperfilm MP (Amersham Biosciences, Amersham, UK) using amplifying screens (Cronex DuPont Lightning Plus, Wilmington, DE, USA).

### 3.5. Construction of P. poae PMA22 Mutant Strains

The knockout strains were constructed by double homologous recombination using the suicide vector pK18mobsacB [31]. To generate the mutant strain *P. poae* ΔmexT, the vector pΔmexT was constructed by synthesizing the 700 bp DNA region at 5′ to the *mexT* gene using the oligonucleotides mexT_UP_F and mexT_UP_R and the 699 pb region at 3′ mexT_DOWN_F and mext_DOWN_R (Table S3). The fragments were digested with the appropriate restriction enzymes and cloned in the unique sites of the pK18mobsacB plasmid. The ligation products were transformed into *E. coli* DH10B-competent cells, and once recombinant candidates were PCR-checked, the cloned region was confirmed by sequencing. The resulting plasmid pΔmexT (Table S2) was used to transform *P. poae* competent cells by electroporation and transformant cells were selected on LB-containing kanamycin (50 µg/mL). Three colonies were picked and cultivated in LB for 24 h. Then, they were spread on plates containing M63P, 10 mM glucose, and 5% (*w*/*v*) sucrose. Colonies unable to grow on kanamycin were selected and analyzed by PCR and sequencing with the appropriate primers (Table S2) to check that the desired mutations were present on the PMA22 genome. To generate the *P. poae* PMA22ΔmexEF-oprN, ~700 pb flanking DNA regions at 5′ and 3′ position were synthesized and cloned into pk18mobsacB using the GeneScript DNA synthesis service.

The pk18mobsacB derivative plasmid pΔmexEF-oprN (Table S2) was transformed by electroporation, followed by the same steps described for the construction of the *P. poae* PMA22 ΔmexT strain.

To generate the spontaneous mutant strains with *nfxC* phenotype, the wild-type PMA22 strain was cultivated on LB agar plates containing Cm (600 µg/mL), and the resistant colonies were sequenced [17].

### 3.6. Complementation of P. poae PAM22 Knockout Strains

Plasmid pSEVA254 from pSEVA collection (http://seva-plasmids.com/, accessed on 1 June 2024) was used as a vector to express the *mexT*, *mexS*, and *mexEF-oprN* genes under the control of the *P_trc_* promoter (Table S2). *mexT* and *mexS* genes were PCR-amplified using *P. poae* PMA22 genomic DNA as a template and MexT_F-MexT-R and MexS_F-MexS_R oligonucleotides, respectively. The resulting PCR fragments were digested and ligated to pSEVA254, generating pmexT and pmexS plasmids. Efflux pump-coding genes *mexEF-oprN* were synthesized and cloned by GeneScript, yielding plasmid pmexEF-oprN.

PMA22::Ptac-mexEF-oprN and PMA22ΔmexEF-oprN::Ptac-mexEF-oprN strains were constructed by tetraparental mating using *E. coli* CC118λpir harboring plasmid pBG_Ptac-mexEF-oprN as a donor, *E. coli* DH5αλpir (pTnS-1) coding the Tn7 transposase as the auxiliary strain, *E. coli* HB101 (pRK600) [32] as the auxiliary helper strain, and *P. poae* PAM22 as the recipient strain. To prepare the recipient strain, 10 mL of late-exponential-phase cultures were centrifuged at 13,000 rpm for 1 min, and the pellet was washed with one volume of sterile 0.85% NaCl solution. The cells were centrifuged again, and the pellet was resuspended to a final volume of 100 μL of 0.85% NaCl solution. One milliliter of overnight cultures of donor and helper strains were centrifuged at 13,000 rpm for 1 min in a microfuge, and the pellet was washed in 500 μL of sterile 0.85% NaCl solution. Fifty microliters of each strain were mixed and pipetted to a 0.22 μm filter disc placed on the NB plate. The plate was incubated overnight at 30 °C. After 6 h, the filter mating disks were collected in a 1.5 mL tube with 1 mL of a sterile 0.85% NaCl solution and vortexed thoroughly to detach the cells from the filter. Afterwards, 100 μL and the rest of the cells were plated on LB plates containing the selective antibiotics kanamycin (50 μg/mL) and gentamycin (50 μg/mL) and screened by PCR using the appropriate primers (Table S2). Selected candidates were grown up to the stationary phase (≈48 h) in LB medium and then plated in LB supplemented with 5% sucrose. The clones that were resistant to sucrose and sensitive to kanamycin were checked by PCR using external primers, and the amplicon was sequenced to confirm the second crossover event.

### 3.7. Production and Extraction of Safracins

Safracins were produced in 50 mL flasks with 20 mL culture medium (MS or M63P) inoculated with the *P. poae* strains to an initial OD_600_ of 0.05 from incubated LB preinoculums for 16 h. After three days, the cultures were centrifuged in a Multispeed Centrifuge CL31R (Thermo Scientific, Waltham, MA, USA) at 3800 rpm for 15 min at 4 °C. The supernatant was transferred to 50 mL falcon tubes and processed for extraction following a protocol adapted from [12]. The pH of the supernatants was adjusted to 9.0, and then a volume of ethyl acetate was added and mixed by vortexing for 1 min. At this point, the sample was centrifuged at 3800 rpm and the aqueous phase was discarded. The sample was evaporated to dryness in a Speedvac centrifuge and solubilized in 100 µL methanol for further analysis.

To analyze safracins inside the cell, 100 mL of culture in M63P medium was inoculated in a 500 mL flask from a preinoculum grown for 16 h in LB medium. After three days, the cells were centrifuged in a Sorval Lynx 6000 (Thermo Scientific) centrifuge (rotor F14-6 × 250y) at 4000 rpm for 20–30 min at 4 °C. The cells were resuspended in distilled water and lysed under high pressure (1000 psi) in a French Pressure Cell Press (American Instruments Company). After centrifuging in a Multispeed Centrifuge CL31R (Thermo Scientific) at 3800 rpm for 15 min at 4 °C, the obtained liquid fraction was subjected to the extraction process described above for further analysis.

### 3.8. Analysis of Safracin Production

Safracin production was analyzed by HPLC-DAD using Agilent Series 1260 Infinity II, Agilent, Santa Clara CA, USA, equipment and monitored at 268 nm. The reversed-phase separation of the metabolites was performed on a C18 column (ZORBAX Eclipse plus C18, 5 µm, 4.6 × 250 mm, Agilent Technologies, Santa Clara, CA, USA) at room temperature and a volume of injection of 20 μL. The mobile phase was: (A) 10 mM ammonium acetate containing 1% diethanolamine (pH 4.0) and (B) acetonitrile [14]. The flow rate was 1 mL/min and the running method was as follows: 20 min 75% A; 35 min gradient up to 100% B; and 40 min gradient to reach 100% A. The data were processed with the software provided by the commercial house itself (OpenLab CDS, Agilent, Santa Clara, CA, USA). The analysis and identification of the safracin intermediates was performed by HPLC-MS. However, the accurate separation of the metabolites produced by *P. poae* and its derivative strains required the use of a solution of 10 mM ammonium acetate containing 1% diethanolamine (pH 4.0) as a mobile phase, which was incompatible with the available HPLC-MS equipment. Therefore, we isolated the species by performing a semipreparative HPLC-DAD (Agilent Series 1260 Infinity II, Agilent, Santa Clara, CA, USA), and the different peaks were manually collected in Eppendorf tubes. Then, HPLC-MS analysis was conducted using a mass spectrophotometry system (Thermo Mod. FinniganTM LXQTM) containing a pump with four separate solvent inputs (Surveyor MS pump Plus), a self-sampling system for multi-sample analysis (Surveyor AS Plus), a DAD (Surveyor PDA Plus), and an LXQ mass spectrophotometer equipped with a linear ion trap. The ionization source used was an ionization electrospray (ESI). The HPLC-MS analysis was performed with a C18 column (ZORBAX Eclipse plus C18, 5 µm, 4.6 × 250 mm, Agilent Technologies, Santa Clara, CA, USA). The mobile phase used was water with 0.1% formic acid (A) and acetonitrile containing 0.1% formic acid (B). The method used was as follows: 5 min gradient from 100% A to 80% A and 20% B; 17 min gradient 75% B and 25% A; 2 min 80% A and 20% B; and 100% A at the end. In the next 2 min, the mixture was inverted, containing 80% A and 20% B, to end with 100% A, balancing the column before the next analysis.

### 3.9. Fluorescence Measurements

A Varioskan Flash (Thermo Scientific) automatic plate reader was used to measure GFP fluorescence and optical density (OD_600_). The cultures were carried out in 20 mL of the corresponding culture medium and incubated for 24 h in agitation. Suitable dilutions of the bacterial culture were prepared to obtain an OD_600_ of 1.0 in 0.85% NaCl, and 200 µL of each cell suspension was added in three technical replicates to a 96-well plate (Falcon). The excitation wavelength was 485 nm and the emission wavelength was 511 nm. The raw data were automatically collected in an Excel sheet. The fluorescence was manually normalized by the actual OD_600_ value of the samples measured in the VarioSkan plate reader. The analysis and representation of the data were carried out with Microsoft Excel.

### 3.10. Bioinformatic Analyses

The Clustal Omega service at https://www.ebi.ac.uk/Tools/msa/clustalo/ (accessed on 1 June 2024) was used to perform multiple protein alignments. The Basic Local Alignment Search Tool (Blast) service at https://blast.ncbi.nlm.nih.gov/Blast.cgi (accessed on 1 June 2024) was used to compare protein sequences. Genome analysis was performed to search for BGCs encoding biosynthetic pathways of secondary metabolites of interest using the online tool antiSMASH 7.0 [33]. Three-dimensional modeling of amino acid sequences requiring structural analysis was performed using the SWISS-MODEL platform (https://swissmodel.expasy.org/, accessed on 1 June 2024). The sequence management (alignment of sequences, genome visualization and plasmid maps, manual search of promoter regions and constructions designs) was carried out using Geneious version 10.0.2 and Prime 2020.0.4 software.

## 4. Discussion

The contiguous location of the *mexT* gene to the BGC sac motivated the search for the nod-box operator regions within the *P_a_* and *P_i_* promoters that drove the expression of the safracin synthetic pathway. The two nod-box sequences recognized by MexT described in the literature were identified in the *P_i_* promoter; however, they were not found in the *P_a_* promoter (Figure 2B). This result suggests that the expression of the *sacABCDEFGHK* and *sacIJ* operons of BGC sac would be controlled by different regulatory mechanisms. The expression data obtained using the transcriptional fusions of *P_a_* and *P_i_* to *gfp*, showed that *P_i_* fusion doubled the expression of *P_a_* in all the tested conditions. Surprisingly, even though *P_a_* did not appear to contain nod-box sequences, MexT showed a specific binding to both *P_a_* and *P_i_* promoters (Figure 3). However, the binding of MexT to promoter regions without nod-box sequences has been described, and thus, according to the in vitro EMSA assay, a limited regulatory role of MexT on the control of the *P_a_* promoter cannot be completely excluded [17,19]. Nevertheless, the identification of nod-box sequences in *P_i_* and the higher binding affinity to this promoter strongly suggest that the expression of the tailoring enzymes is precisely controlled by MexT in the safracin pathway.

The *mexS/mexT* regulatory system has been described in *P. aeruginosa* as a mutational “hot spot” [25]. Strains with mutations in these two genes show the *nfxC* phenotype, which promotes the induction of the MexEF-OprN transport system, increases the resistance to antibiotics, promotes faster growth in the early exponential phase, and modulates virulence [21,22,26]. The functional nature of the mutations that cause this phenotype allows its selection by exposing the cells to high concentrations of chloramphenicol (600 µg/mL). Our results showed that the *mexT* and *mexS* genes of *P. poae* PMA22 were fully functional, and this allowed us to select different *nfxC* mutants resistant to chloramphenicol. Three randomly selected *P. poae* PMA22 strains with the nfxC phenotype showed that the mutations were always located between nucleotides 705 and 780, encoding the cofactor-binding domain of *mexS* (Figure 3). These results suggest that the pleiotropic character of the *nfxC* phenotype has a channeling effect on the selective pressure towards *mexS*. In this region, residue Gly238 is highly conserved [27], suggesting that the main target of the selective pressure that generates the *nfxC* phenotypes could be this residue.

In vivo experiments confirmed that the overexpression of *mexT* and *mexS* had no significant effects on the *P_a_* promoter (Figure 5B,C). Significant differences were not observed in the expression of *P_a_* in the PMA22ΔmexT mutant strain (Figure 5D), nor in the PMA22NfxC1 strain that contained a non-functional *mexS* gene. The results obtained in the in vitro EMSA assay might suggest that the regulation of the Pa promoter could be mediated by alternative systems that prevail over the action of MexT, or, perhaps, that the low affinity binding of MexT to the *P_a_* promoter detected in vitro might not be functional in vivo. In contrast, the overexpression of *mexT* had a significant activating effect on the expression of the *P_i_* promoter (Figure 5B,C), confirming the regulatory role of MexT on the BGC sac expression. However, the activating effect of MexT on the *P_i_* promoter in the heterologous system of *E. coli* DH10B was superior to that observed in PMA22. This finding does not allow us to rule out that the expression of *P_i_* could be controlled by additional regulatory mechanisms in PMA22. The mutant PMA22ΔmexT (pPi, pSEVA254) presented two-fold lower *P_i_* expression than the PAM22 (Figure 5D), indicating that half of the *P_i_* expression level is maintained by MexT activation. On the other hand, over-expression of MexS significantly decreased the expression of the *P_i_* promoter in *P. poae* and *E. coli* strains, confirming its role as a repressor.

Since *P_i_* drives the expression of safracin-tailoring enzymes, the regulatory effect of MexT would be expected to have an impact on the efficiency in the processing of intermediates P19A, P19B, P22A, and P22B, and, therefore, an improvement in the production of safracin (Figure 1). The production of analogs and safracin of the strains supported this hypothesis. We observed that the PMA22ΔmexT strain accumulated more intermediates and that the synthesis of safracin was lower than that obtained with PMA22 (Figure 6 and Figure S2B). The PMA22NfxC1 strain, carrying a non-functional MexS, produced slightly more safracin than the wild-type strain PMA22 and showed a lower accumulation of intermediates. Over-expression of *mexS* caused a decrease in the production of safracins and a large accumulation of intermediates (Figure 6 and Figure S2B). However, overexpression of *mexT* did not have a positive effect on the production, although an increase in the expression of *P_i_* was observed in the PMA22 (pPi, pmexT) strain. Due to the pleiotropic nature of MexT, its overexpression could be involved in other mechanisms independent of the expression of the BGC sac promoters, which could be indirectly affecting safracin production.

The MexT/MexS regulatory tandem modulates the flow of reactions carried out by SacJ and SacI tailoring enzymes, whose expression is directed by the *P_i_* promoter. In this sense, different mechanisms have been described by which BCGs evolve to synthesize greater diversity of bioactive compounds, which provides a competitive advantage in producing microorganisms. One of the strategies that evolution has found to generate this diversity consists of post-NRPS modifications based on the action of tailoring enzymes [34,35]. Although, in most cases, this diversity occurs between BGCs present in different bacteria, the independent regulation of the tailoring enzymes in the case of BGC sac could provide a mechanism to generate safracin analogues in different proportions according to the expression of *P_i_*, contributing to provide a greater diversity of active compounds in PMA22.

To confirm the participation of MexEF-OprN in the transport of safracin, we constructed the mutant strain PMA22ΔmexEF-oprN, which secretes less safracin to the extracellular medium and retains more safracin intracellularly compared to PMA22 (Figure 7B). Interestingly, by reducing the percentage of mannitol in the M63P medium from 8% to 4%, the PMA22ΔmexEF-oprN mutant strain produced only 2.4-fold less safracin than PMA22, suggesting a relationship between osmotic stress and safracin transport.

A divergent effect was demonstrated with the PMA22::Ptac-mexEF-oprN strain, which contains a second copy of the MexEF-OprN transport system integrated into the chromosome under the control of the IPTG-inducible *P_tac_* promoter. This strain secretes 4 times more safracin than PMA22. This result suggests that MexEF-OprN is involved in safracin transport and that safracin transport plays a key role in the increased safracin production observed under eliciting conditions. Our results suggest that an increased expression of mexEF-oprN favors the secretion of safracin, thus avoiding the intracellular accumulation of safracin and its intermediates, which could repress the biosynthetic process. There are several examples in the literature in which the synthesis of secondary metabolites is autoregulated through the inhibition of enzymes of the biosynthetic pathway by the accumulation of precursors or final products of the same pathway [36,37].

On the other hand, it is worth mentioning that multiple genes capable of regulating the expression of *mexEF-oprN* in *P. aeruginosa* have been described, such as *ampR* [38], *mvaT* [39], *mxtR* [40], *cmrA* [41], *brlR* [42], the *parR/parS* two-component system [43], and the *pvcB* gene [44]. Leaving aside the *brlR* and *pvcB* genes that are not present in the PMA22 genome, the other orthologous genes can be postulated as possible regulators of safracin production.

Our results suggest that MexT coordinates the safracin post-NRPS tailoring modifications and transport in *P. poae* PMA22. In this sense, Jain and Cox proposed that the coordination of the synthesis of secondary metabolites with their transport could be an extended mechanism in the biosynthesis of NRPs and polyketides [45]. The synthesis of the polyketide phthiocerol dimycocerosate (PDIM) is precisely synchronized with its secretion through an RND-type transporter that would be physically coupled with the polyketide synthase. Landomycin A biosynthesis in *Streptomyces cyanogenus* S136 represents another model of coordination of bioactive molecule synthesis with its transport [46]. The TetR-type LanK regulator, belonging to the landomycin A BGC, has been shown to repress the synthesis of precursor-modifying enzymes late in the pathway, as well as genes responsible for landomycin transport [46]. The accumulated precursors bind to LanK, inducing the synthesis of the final product, landomycin A (LaA), and transport. Therefore, the main biological role of LanK appears to be the inhibition of the premature extrusion of early LaA precursors from cells, which in turn creates the optimal conditions for the accumulation of LaA as the major landomycin in *S. cyanogenus* S136 [46].

The coordination of synthesis and transport could be energetically favorable while promoting specificity and directionality in transport processes [45], providing PMA22 a competitive advantage. In addition, the coordination by MexT of the induction of Pi and the transport of safracins ensures that the final products are exported upon completion of the synthesis. Another important aspect is that safracin B is more cytotoxic than the intermediates of the pathway [47]. This could be the reason why the coordination of the tailoring reactions, which transform the intermediates into the final product, and the transport of the final compound to the extracellular medium could be adaptive mechanisms of the cell to avoid the accumulation of a toxic compound inside the cell.

## Data Availability

All data generated or analyzed during this study are included in this published article (and its Supplementary Materials file).

## Supplementary Materials

The following supporting information can be downloaded at: https://www.mdpi.com/article/10.3390/md22090418/s1, Figure S1: Hyper-production and purification of MexT regulator. (A) Cloning of the *mexT* gene into the pET29a(+) expression vector. The pETmexT_F/R oligonucleotides were used to PCR-amplify the *mexT* gene. Plasmid pETmexT allows the expression of a MexT-(His)_6_ fusion protein under the control of LacI^q^ in *E. coli* BL21 (DE3). (B) SDS-PAGE gel (12.5%) of the purified fractions of MexT-(His)_6_ fusion protein using a Ni-NTA affinity chromatography. Lane E: protein extract excluded on the affinity column. Lanes 20, 50, 100, 100, 250, 500, and 750 represent the fractions obtained after the successive washes of the affinity column by adding 20 mM to 750 mM imidazole to elute the MexT-(His)_6_ protein. Figure S2: (A) Mass spectrometric identification of the chromatographic profile of safracin production in *P. poae* PMA22. The chromatogram corresponds to the strain grown on MS medium with 4% mannitol. The peaks indicated in the chromatogram were isolated by semi-preparative HPLC analysis and the chemical species were identified by mass spectrometry, as shown in the upper part of the figure based on the previous data [47]. (B) A comparative chromatogram in three blocks shows the effect of MexT and MexS on the efficiency of safracin production. The first chromatograms show the effects of MexT and MexS when overexpressed in trans in PMA22 strains. The second chromatogram shows the production compound profiles of PMA22, PMA22ΔmexT, and PMA22NfxC1 strains. The quantification of the areas is detailed over the peaks previously identified by mass spectrometry; Table S1. Strains used in this work; Table S2. Plasmids used in this work; Table S3; Primers used in this work.
