# Supplementary material for: Regulation of Safracin Biosynthesis and Transport in Pseudomonas poae PMA22
Source: Mar Drugs. 2024 Sep 13;22(9):418. doi: 10.3390/md22090418 (PMC11432991; doi:10.3390/md22090418)
Supplement: Supplementary file 1 [file marinedrugs-22-00418-s001.zip › marinedrugs-3147605-supplementary.pdf]

Supplementary file

Regulation of safracin biosynthesis and transport in *Pseudomonas poae* PMA22

J. Gerardo Hernández Delgado[1], Miguel G. Acedos[1], Fernando de la Calle[2], Pilar Rodríguez[2], José L. García[1] and Beatriz Galán[1,*]

[1] Department of Microbial and Plant Biotechnology, Centro de Investigaciones Biológicas Margarita Salas, Agencia Estatal Consejo Superior de Investigaciones Científicas (CSIC), Madrid, Spain.
[2] Research and Development Department, PharmaMar S.A., Madrid, Spain.
* Correspondence: bgalan@cib.csic.es

Corresponding author: bgalan@cib.csic.es

List of tables and figures

Table S1. Strains used in this work.

Table S2. Plasmids used in this work

Table S3. Primers used in this work

Figure S1. Hyper-production and purification of MexT regulator. A) Cloning of the *mexT* gene into the pET29a(+) expression vector. The pETmexT_F/R oligonucleotides were used to PCR-amplify the *mexT* gene. Plasmid pETmexT allows the expression of a MexT-(His)6 fusion protein under the control of LacI^{q} in *E. coli* BL21 (DE3). B) SDS-PAGE gel (12.5%) of the purified fractions of MexT-(His)6 fusion protein using a Ni-NTA affinity chromatography. Lane E: protein extract excluded on the affinity column. Lanes 20, 50, 100, 100, 250, 500 and 750 represent the fractions obtained after the successive washes of the affinity column by adding 20 mM to 750 mM imidazole to elute the MexT-(His)6 protein.

Figure S2. A) Mass spectrometric identification of the chromatographic profile of safracin production in *P. poae* PMA22. The chromatogram corresponds to the strain grown on MS medium with 4% mannitol. The peaks indicated in the chromatogram were isolated by semi-preparative HPLC analysis and the chemical species were identified by mass spectrometry, as shown in the upper part of the figure. B) A comparative chromatogram in three blocks shows the effect of MexT and MexS on the efficiency of safracin production. The first chromatograms show the effect of MexT and MexS when overexpressed in *trans* in PMA22 strains. The second chromatogram shows the production compound profile of PMA22, PMA22ΔmexT and PMA22NfxC1 strains.

The quantification of the areas is detailed over the peaks previously identified by mass spectrometry.

Table S1.

Strains	Genotype/Phenotype	Reference
Escherichia coli		
DH10B	F⁻, Δ(*ara-leu*)*7697*, Δ*(lac)X74*, *galK* φ80d*lacZΔM15*, *recA1*, *endA1*, *rpsL*, Δ*(mrr-hsdRMS-mcrBC)*, λ⁻, *deoR*, *galU*, *nupG*	Invitrogen
BL21(DE3)	F⁻ *ompT gal dcm lon hsdS*$_B$($r_B^-m_B^-$) λ(DE3 [*lacI lacUV5-T7p07 ind1 sam7 nin5*]) [*malB*⁺]$_{K-12}$(λ^S)	(Green et al., 2012)
S17-1λpir	Tp^R, Sm^R, *recA*, *thi*, *hsdRM*⁺, *RP4-2-Tc::Mu::Km*, *Tn7*, *λpir lysogenic*	(de Lorenzo and Timmis, 1994)
Pseudomonas poae		
PMA22	Wild type	PharmaMar
PMA22NfxC1	PMA22 derivative coding a nonfunctional *mexS* gene due to a 14 bp deletion from position 237	This work
PMA22NfxC2	PMA22 derivative coding a nonfunctional *mexS* gene due to a 12 pb deletion from position 237	This work
PMA22NfxC3	PMA22 derivative coding a nonfunctional *mexS* gene due to an insertion of 32 bp at position 256	This work
PMA22Δ*mexT*	PMA22 derivative with a deletion on *mexT* gene	This work
PMA22Δ*mexEF-oprN*	PMA22 derivative with a deletion on *mexEF-oprN* genes	This work
PMA22::*Ptac-mexEF-oprN*	PMA22 derivative carrying an insertion in the chromosome coding the MexEF-OprN efflux pump under the control of P_{tac} promoter	This work
PMA22Δ*mexEF-oprN*::*Ptac-mexEF-oprN*	PMA22Δ*mexEF-oprN* derivative carrying an insertion in the chromosome coding the	This work

	MexEF-OprN efflux pump under the control of P_{tac} promoter	
PMC-3	PMA22 derivative safracin hyperproducer	PharmaMar

Table S2.

Plasmids	Description	References
pTnS-1	Amp^R, *ori*-R6K, containing *tnsABC-tnsD* operon	(Choi et al., 2005)
pRK600	Cm^R, *ori*-ColE1, RK2 Mob^+, Tra^+	(Knudsen and Karlstrom, 1991)
pSEVA637	Gm^R, *ori*-pBBR1, *gfp*.	https://seva-plasmids.com/
pP*a*	pSEVA637 derivative carrying the P_a promoter región of BGC *sac* from *P. poae* PMA22	This work
pP*i*	pSEVA637 derivative carrying the P_i promoter región of BGC *sac* from *P. poae* PMA22	This work
pSEVA254	Km^R, *ori*-RSF1010, P_{trc}	https://seva-plasmids.com/
p*mexT*	pSEVA254 derivative containing *mexT* gene	This work
p*mexS*	pSEVA254 derivative containing *mexS* gene	This work
p*mexEF-oprN*	pSEVA254 derivative containing *mexEF-oprN* operon	This work
pET29a(+)	Km^R, Cloning and expression vector, *ori*-ColE1, P_{T7}	Novagen
pET29*mexT*	pET29a(+) derivative containing the *mexT* gene	This work
pK18mob*sacB*	Km^R, *ori*-ColE1, Mob^+, *lacZα*, *sacB*; vector for allelic exchange homologous recombination mutagenesis	(Schäfer et al., 1994)
pΔ*mexT*	Km^R, pK18mobsacB derivative containing fragments UP and DOWN flanking *mexT* gene,	This work
pΔ*mexEF-oprN*	Km^R, pK18mobsacB derivative containing fragments UP and DOWN flanking *mexEF-oprN* operon	This work

pBG	Km^R, Gm^R, *ori*-R6K, Tn7L and Tn7R extremes, BCD2–msfgfp fusion	Zobel et al., 2015
pBG_*Ptac-mexEF-oprN*	Km^R, Gm^R, pBG derivative carrying *mexEF-oprN* operon flanked by Tn7L and Tn7R	This work
pCR-Blunt II-TOPO	Km^R, Neo^R, PCR cloning vector, *ori*-pBR322	Thermofisher

Table S3.

Primer	Sequence[1] (5'→3')	Use[2]
mexT_F	GAATCTCTAGATGACCTAAGGAGGTAAATAATGAACCGTAACGACCTGC	Amplification of *mexT* for cloning in pSEVA254 (*Xba*I/*Hin*dIII)
mexT_R	TTAGCAAGCTTTTAAAGGCTATCGGGGTCGC	
pETmexT_F	ATCGAACATATGATGAACCGTAACGACCTGC	Amplification of *mexT* for cloning in pET29a(+) (*Nde*I/*Hin*dIII)
pETmexT_R	GACTGGAAGCTTAAGGCTATCGGGGTCGC	
mexS_F	GGCATAGAATTCTGACCTAAGGAGGTAAATAATGTCCCGCACGATCCGTTT	Amplification of *mexS* for cloning in pSEVA254 (*Eco*RI/*Hin*dIII)
mexS_R	ACCGATTAAGCTTGGTTTGGAACACTCCTTTGCG	
Pa_F	TTAGCATGCCTTGACTTTTAATCTGAACGGGCA	Amplification of 220 pb covering *Pa* promoter for cloning in pSEVA637 (*Sph*I/*Hin*dIII).
Pa_R	TTGACGTAAAGAAGCTTGCACC	

Pi_F	TTAGCATGCCCCCGAAATGATGGACAAGGA	Amplification of 240 pb covering P_i promoter for cloning in pSEVA637 (*Sph*I/*Hin*dIII)
Pi_R	GGCAAGCTTTGAACATCCATATATCAACAGTTATCC	
mexT_UP_F	TTAGAGAATTCTCAGCAGCGGCGTGTAGTGC	Amplification of ~600 pb fragment upstream and downstream *mexT* for cloning in pk18mobsacB (*Eco*RI/ *Bam*HI/*Sph*I)
mexT_UP_R	TGAACTGGATCCCCTGGAAGAGTCAGCTTGCT	
mexT_DOWN_F	TGAACTGGATCCGACCCCGATAGCCTTTAATT	
mexT_DOWN_R	CGAATTGCATGCTGCTTTTCCGATTCAATACG	
mexS_Sec_F	GGTTACTTTGACGAGGTTTGCG	*mexS* sequencing
mexS_Sec_R	GGGTTTGGAACACTCCTTTGC	
ΔmexT_Sec_F	CAAATTGCCCAGGCTTGACC	*mexT* sequencing
ΔmexT_Sec_R	AGGTTTGCAGATGGGGATCG	
ΔmexEF-oprN_Sec_F	GTCTTTGGGGCTCAGGTCC	Cheking *mexEF-oprN* deletion
ΔmexEF-oprN_Sec_R	CGCAGCAAGGCAACCTCG	

[1]Restriction sites are underlined. [2]Restriction enzymes used for cloning are indicates in brackets.

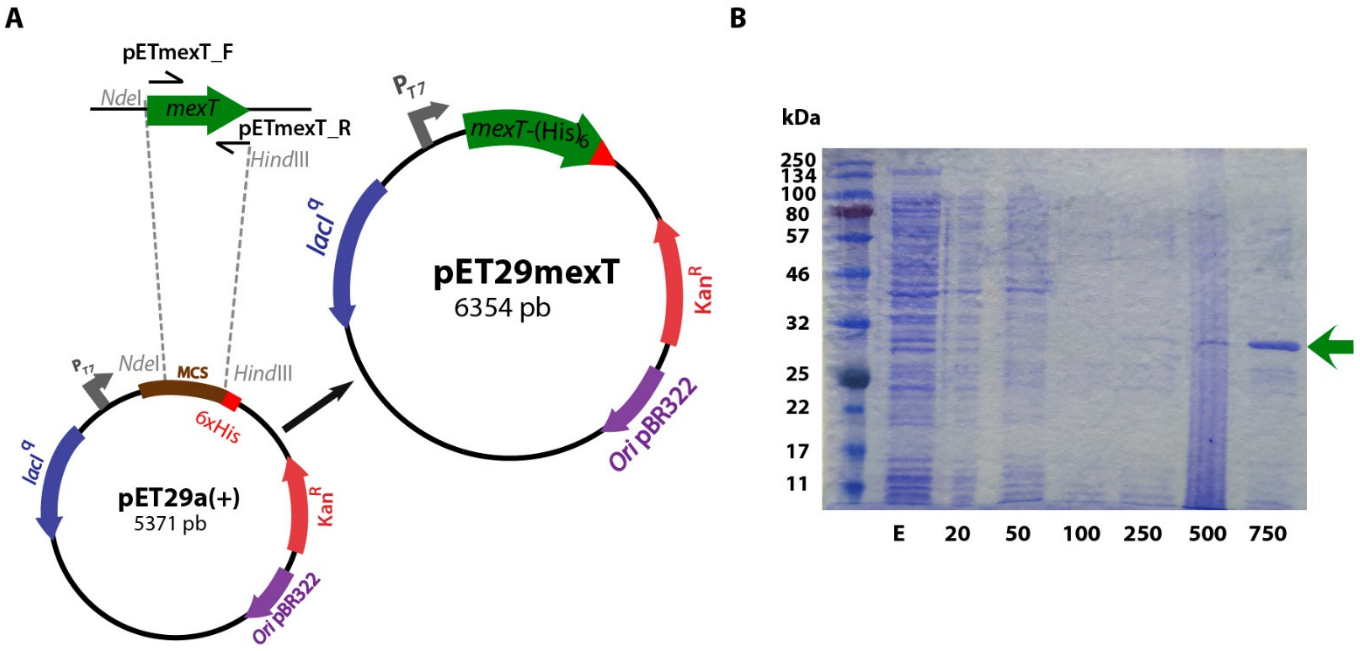
A
pETmexT_F
NdeI
mexT
pETmexT_R
HindIII
PT7
mexT-(His)6
lacIq
pET29mexT
6354 pb
KanR
Ori pBR322
PT7
NdeI
MCS
HindIII
6xHis
lacIq
pET29a(+)
5371 pb
KanR
Ori pBR322
B
kDa
250
134
100
80
57
46
32
25
22
17
11
E
20
50
100
250
500
750

Fig S1.

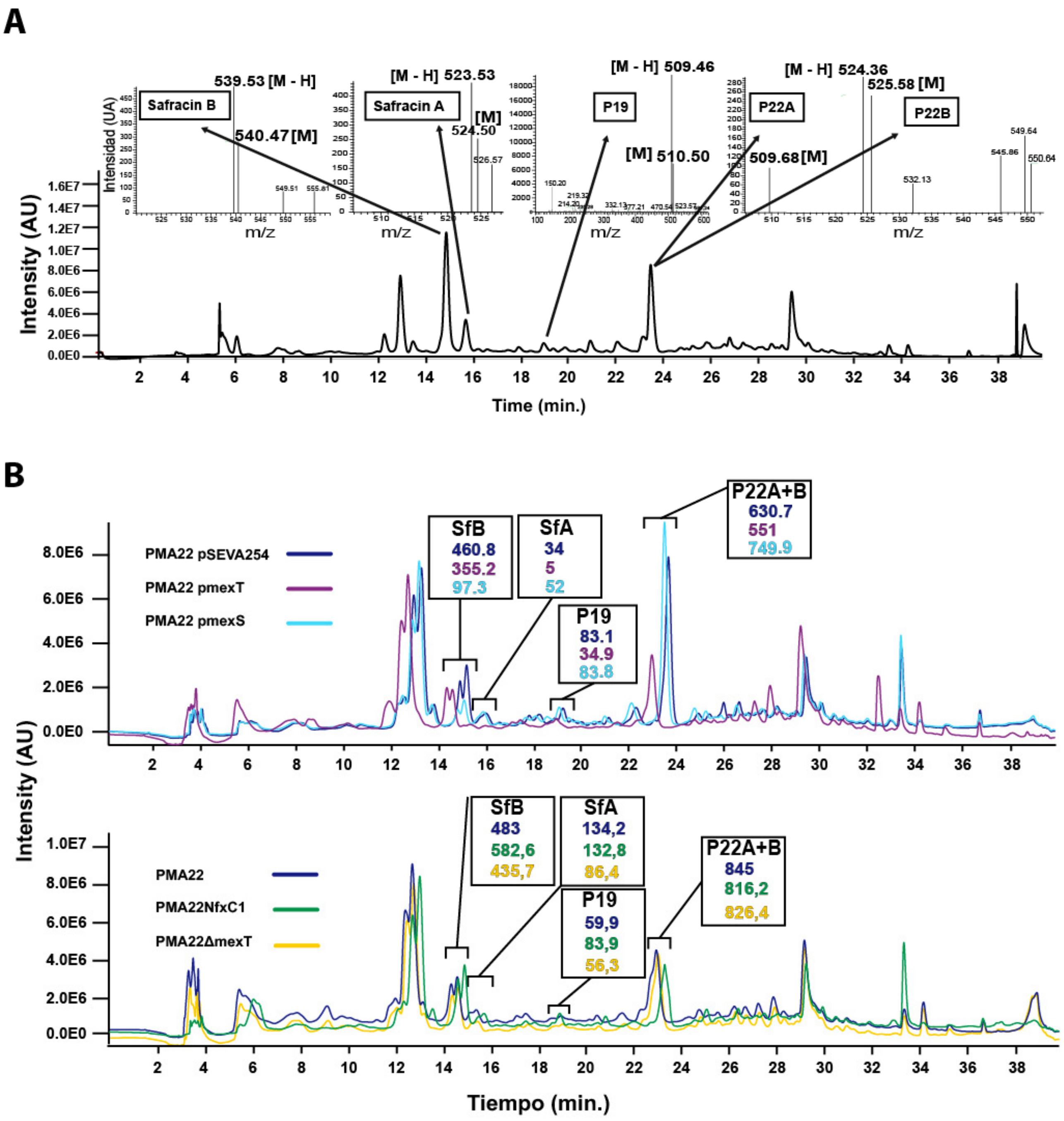
A
Safracin B
539.53 [M - H]
540.47 [M]
Safracin A
[M - H] 523.53
[M]
524.50
P19
[M - H] 509.46
[M] 510.50
P22A
509.68 [M]
[M - H] 524.36
525.58 [M]
P22B
Intensity (AU)
Time (min.)
B
PMA22 pSEVA254
PMA22 pmexT
PMA22 pmexS
SfB
460.8
355.2
97.3
SfA
34
5
52
P19
83.1
34.9
83.8
P22A+B
630.7
551
749.9
PMA22
PMA22NfxC1
PMA22ΔmexT
SfB
483
582,6
435,7
SfA
134,2
132,8
86,4
P19
59,9
83,9
56,3
P22A+B
845
816,2
826,4
Tiempo (min.)

Figure S2.
